# Neuroethics with regard to treatment limiting and withdrawal of nutrition and hydration in long lasting irreversible full state Apallic Syndrome and Minimal Conscious State

**Published:** 2008-11-15

**Authors:** von Wild Klaus

**Affiliations:** * Department of Biochemistry, Faculty of Medicine, University of Medicine and Pharmacy “Carol Davila”

## Abstract

*Introduction:* Epidemiology in Europe shows constantly increasing figures for the Apallic Syndrome (AS)/Vegetative State (VS) as a consequence of advanced rescue, emergency services, intensive care treatment after acute brain damage, and high standard activating home nursing for completely dependent end stage cases secondary to progressive neurological disease. Management of patients in irreversible apallic syndrome has been the subject of sustained scientific and moral-legal debate over the last decade.

*Methods: *Neuroethics coming more and more into consideration when neurological societies address key issues relating to AS/VS prevalence and quality management. With regard to treatment limiting and withdrawal of nutrition and hydration of patients suffering from irreversible full state Apallic Syndrome and Minimal Conscious State.

*Results:* The overall incidence of new AS/VS full stage cases all aetiology is 0.5 - 2/ 100.000 population per year. About one third is traumatic and two thirds are non-traumatic cases. The worst prognosis might be expected from non-traumatic hypoxemic apallic syndrome. The main conceptual criticism is based on assessment and diagnosis of all different AS/VS stages based solely on behavioural findings without knowing the exact or uniform pathogenesis or neuropathologic findings. No special diagnostics, no specific medical management can be recommended for class II or III AS treatment and rehabilitation. But in United Kingdom, The Netherlands, Belgium, and Switzerland active euthanasia is now practiced in medicine taking into account the uncertainty of the right diagnose the clinical features for humanistic treatment of patients in irreversible *“AS full or early , remission stages*” .

*Discussion:* As long as there is no single AS/VS specific diagnostic tool, no specific laboratory investigation regimen to be recommended neuroethical principles demands by all means a humanistic (ethical) activating nursing even in the irreversible full stage AS cases. Full acceptable is only the palliative pain therapy with renunciation of maximal therapy.

Active euthanasia is a criminal act and has to be respected as such in neuroscience.

## Objective

In following the Helsinki Declaration of the World Medical Association, published in 1964 and its numerous amendments [**1**], doctors are obliged to use all available means to help their patient and to leave nothing untried. There is main demand that is based on a broad consensus among doctors, other medical and rehabilitation professionals, and next of kin that medical and rehabilitative treatment interventions and adequate activating nursing of adults and children who remain in an AS full stage or in an AS early remission phase 2 and 3 (**Table 1**) for 6 to 12 months should be provided based on need with every effort to maintain comfort, eliminate complications and optimize functioning and recovery [**5**-**9**, **20**, **21**,**30**, **31**, **34**]. This agreement of physicians is following the Hippocratic Oath despite divergent statements of national and international ethical consensus conferences and diametrically opposed national penal laws and penal decisions in respect with treatment-limiting and withdrawal of nutrition and hydration [**2**-**19**, **21**,**22**,**25**,**29**,**38**]. For more details we would like to refer the interested reader to the critical reviews by Jennett [**17**], Gigli and Valente [**14**], and Shewmon [**2**].

## Methods

*Apallic syndrome full stage* [**8**, **9**] is a separate entity characterized by the classical symptoms and signs of *wakefulness without awareness of self and environment* and related to signs and symptoms of functional disinhibition of the upper brainstem. Clinical assessment of AS patients requires additional, advanced training and at least three years of personal expertise in practical AS management to be able to make the right AS diagnosis. 

**Table 1:** ***Gerstenbrand‘s Innsbruck- AS*** remission Scale for individuals emerging from AS full stage (1977)

1. Optical fixation, primitive emotional reactions minimal differentiated, motor primitive patterns diminished.
2. Optical tracking; differentiation of emotional reactions, aim directed on motor primitive patterns, mass movements partly directed, diminishing of the flexed stretch position, spastic and Parkinson’s symptoms observed
3. Obeying simple commands; emotional reactions responding to the situation (positive and negative), organised primitive motor patterns; grasping reflex and oral reflexes (initial Klüver Bucy symptoms); patient accepts oral feeding; beginning of finalized movements,
4. Klüver Bucy full Syndrome: grasping, bringing the object to the mouth without recognizing the object, interest in the genital region, react to simple orders, primitive sounds.
5. Post-Klüver Bucy phase: residual arm flexion and lower limb flexion-extension movements of head, trunk and extremities become more finalised, simple words are produced,
6. Korsakov Syndrome, somnolence, residual spasticity and extrapyramidal symptoms and primitive motor patterns present, first going practised
7. Amnesic psychosyndrome, residual motor disturbances, residual primitive motor patterns.
8. Organic psychosyndrome with local and diffuse neurological deficits.
**Nota Bene:** The defect (end) stage is characterized by more or less local, regional and diffuse deficits of higher brain functioning

Detailed clinical physical and psychological cognitive assessment and expertise are requested to be of utmost importance grading the reported high level of misdiagnosis in the literature as to avoided false and even fatal misdiagnosed treatments [**1**,**3**,**4**,**12**,**21**,**27**,**31**,**36**-**39**] Careful clinical evaluation, however, needs a considerable amount of time, measured in weeks rather than hours, and during different day times, if varying levels of function are to be identified and correctly classified on a special questionnaire. The ability to generate a behavioural response fluctuates from day to day, hour to hour, and even from minute to minute. Sampling of spontaneous behaviour, structured intervals with the aid of care staff and/or relatives is important. Continuous documentation of patient’s functioning on special charts, of all interventions, laboratory tests, medication, and secondary complications is essential.

For both the physical and the mental-cognitive neurobehavioral assessment [**28**-**35**], we recommend the German Coma Remission Scale (**CRS**). The best performance of the CRS Score when it is assessed by all team members has been demonstrated to be reliable and efficient. At present, CRS is one of the best assessment tools able to detect even subtle changes and fluctuations in neurobehavioral abnormalities (**Table 2**). CRS became scientifically widely accepted and is therefore now used daily in all German speaking European countries during intensive care and early rehabilitation in neurosurgery and neurology [**39**]. **SMART** (*Sensory Modality Assessment and Rehabilitation Technique*) was established in the UK and is widely used in there and in some other English speaking parts of Europe. It was used as a reliable and efficient method of choice to detect misdiagnosed AS patients when as many as 43% of AS patients thought to be in the vegetative state were actually aware [**31**]. 

**Table 2:** **German Coma Remission Scale **(*CRS*) (German Task Force of Neurological-Neurosurgical Early Rehabilitation 1993/2000

Table 2.1.		
Front page	Patient’s name:	
Date:		
Investigator (initials):		
**1. Arousability/attention **(to any stimulus)		
Attention span for 1 minute or longer	5	
Attention remains on stimulus (longer than 5 sec)	4	
Turning towards a stimulus	3	
Spontaneous eye opening	2	
Eye opening in response to pain	1	
None	0	
**2. Motoric response** (minus 6 points from max. attainable sum if tetraplegic)		
Spontaneous grasping (also from prone position)	6	
Localized movement in response to pain	5	
Body posture recognizable	4	
Unspecific movement in response to pain (vegetative or spastic pattern)	3	
Flexion in response to pain	2	
Extension in response to pain	1	
None	0	
**3. Response to acoustic stimuli (e.g. clicker)** (minus 3 points from max. attainable sum if deaf)		
Recognizes a well-acquainted voice, music, etc.	3	
Eye opening, turning of head, perhaps smiling	2	
Vegetative reaction (startle)	1	
None	0	
**4. Response to visual stimuli** (minus 4 points from max. attainable sum if blind)		
Recognizes pictures, persons, objects	4	
Follows pictures, persons, objects	3	
Fixates on pictures, persons, objects	2	
Occasional, random eye movements	1	
None	0	
5. Response to tactile stimuli		
Recognizes by touching/feeling	3	
Spontaneous, targeted grasping (if blind), albeit without comprehension of sense	2	
Only vegetative response to passive touching	1	
None	0	
**6. Logomotor (speech motoric) response** (tracheotomy = 3 in case lips can be heard to utter guttural sounds/seen to mime „letters”)		
At least one understandably articulated word	3	
Unintelligible (unarticulated) sounds	2	
Groaning, screaming, coughing (emotional, vegetative tinged)	1	
No phonetics/articulation audible/recognizable	0	
Sum score:		
Max. Attainable score (of 24) for this patient		
Table 2.2		
Back page guidance		
1. Arousability/attention		
5 pts: Patient can direct his/her attention towards an interesting stimulus for at least 1 minute (perceivable by vision, hearing, or touching; stimulus: persons, objects, noises, music, voices, etc.) without being diverted by secondary stimuli.		
4 pts: Attention fixed to a stimulus for a discernible moment (fixation with the eyes, grasping, and feeling or „pricking up of ears”); patient is, however, easily diverted or „switches off„.		
3 pts: Patient turns to source of stimulus by moving eyes, head, or body; patient follows moving objects. Vegetative reactions should also be observed (patient capable only of vegetative reaction).		
2 pts: Spontaneous opening of eyes without any external stimulus, e.g. in connection with a sleep-waking-state rhythm.		
2. Motoric response		
6 pts: Patient spontaneously grasps hold of held-out everyday objects (only if patient’s vision function is intact, otherwise lay object on back of patient’s hand). OR patient able to respond to such gestures with an invitational character only with a delay or inconsistently, yet adequately, due to paralysis or contraction.		
* Note regarding the following items (use of pain stimuli):The pain stimuli must be applied to the various limbs and to the body trunk, since there may be regional stimulus-perception impairments; pain stimuli can take the form e.g. of a gentle twisting pinch of a fold of skin, pressure applied to a fingernail fold, tickling of the nose.*		
5 pts: Patient responds to pain stimuli defensively after localization, by a targeted and adequate measure, e.g. pushing away, sweeping motions of the hand, etc.		
4 pts: The patient should be seated upright: tests for the sense of balance and/or posture by slight pushes applied to the body (corrective movements of trunk or extremities).		
3 pts: Untargeted withdrawal from pain stimulus or merely vegetative reactions (tachycardia, tachypnea, agitation) or increase of spastic pattern.		
2 pts: Strong, hardly resolvable flexion, especially in the arms/elbows. Legs may stretch out.		
1 pt: Typical „decerebrate rigidity” with spastic extension of all extremities, in many cases opisthotonus (dorsal overextension/hyperlordosis).		
3. Response to acoustic stimuli (tests as a rule to be carried out beyond patient’s field of vision!)		
3 pts: Patient can recognize voices or music, i.e. he/she is able either to name the stimulus or to react in a differentiated manner (e.g. to certain pieces of music or persons with plea¬sure or defensively).		
2 pts: Patient only opens eyes, fixates or turns to source of stimulus with his/her head, in some cases accompanied by emotional expressions such as smiling, crying.		
1 pt: Rise in pulse and/or blood pressure, perspiration or agitation, excessive twitching of the body		
1 pt: Rise in pulse and/or blood pressure, perspiration or agitation, excessive twitching of the body, slight triggering of eye blinking.		
*Note:* Similar to the procedures applied when testing the motor responsiveness by the appli-cation of pain stimuli, the use of a clicker held directly next to each of the patient’s ears (bilateral testing) suggests itself as the relatively strongest non-pain-involving stimulus for items 1 and 2; if the response is positive, the patient can be assumed to still be in possession of his/her hearing and the stimuli can be made more manifold.		
4. Response to visual stimuli (must be presented without speaking or any other form of comment)		
4 pts: Patient recognizes pictures, objects, portraits of familiar persons.		
3 pts: Follows pictures etc. with the eyes without any sign of recognition or questioning, in-consistent recognition.		
2 pts: Fixates moving pictures or objects without being able to follow them properly, or when picture/object moves outside patient’s field of vision patient makes no attempt to keep track.		
5. Response to tactile stimuli		
3 pts: Patient capable of feeling and recognizing objects, hands of other persons, etc. even if his/her sense of vision is absent and the objects must be placed on the skin/in the hands; adequate response to stimuli in the area of the mouth/face (edible/inedible, e.g. response to a kiss).		
2 pts: Touches, feels, and grasps targeted, but without an adequate reaction.		
1 pt: Unspecific response to stroking and touch (vegetative signs such as agitation, raised pulse).		
6. Logomotor (speech motor) response		
3 pts: Patient is capable of expressing an intelligible word, even if this is not related to the context or situation. Names also count as words here.		
2 pts: Patient utters unintelligible sounds, e.g. slurred, also repetition of syllables or similar („ma-ma”, „au”.		
**Total score:** In the event that certain channels of sense or motor systems are completely absent („blind”, „deaf”, „plegic”), the point scores of the respective category must be sub¬tracted from the maximum attainable score, e.g. 12/21 points instead of 12/24 points.		

For later recovery states the Early Barthel Index (EBI) and the Functional Independence Measure (FIM) have shown complementary results.

The public and societies at least Europe should accept the „*social economic burden*” itself to supporting the next of kin and relatives taking care of the patient financially, morally, and psychologically instead to pretend to have a right on human life [**5**-**7**,**38**-**40**], That, however, happened now for example in The Netherlands [**19**] Belgium, and Swiss. However, the inevitably dilemma we are confronted with is the ongoing confusion between personally experienced or even suggested quality of life of a person in the apallic full stage or one of the early but not changing remission stages and the inherent dignity of every human being, calling for respect of his/her rights and in respect with his/her personally signed decision of free will that should be final concerning withdrawal of treatment when seriously ill without any chance and hope for recovery to live a human social life again [**1**,**4**,**7**,**12**,**13**,**15**,**19**,**21**,**22**,**25**,**29**,**40**]. The Paris Declaration from September 2005 gives additional details especially concerning the right of patient unable to consent.

**Table 3:** CRS scores when assessed in 175 TBI at admission for neurorehabilitation with special remarks on low scores in early neurosurgical rehabilitation for MS

CRS at admission	Patients admitted for rehabilitation N=258			Patients admitted for ENNR N=100		
	MS	H	Else	MS	H	Else
< 10	14	7	4	14	2	3
9-19	10	24	3	9	9	1
20-23	7	12	5	7	3	1
=>24	35	29	25	32	2	2
Total	66	72	37	63	16	7
No data	2	10	71	1	16	13
Patients	68	82	108	64	16	20

## Results

The overall incidence of new AS/VS full stage cases of all aetiology is 0.5-2/100.000 population per year. About one third is traumatic and two thirds are non-traumatic cases.

In our prospective clinical multiple centre analysis of quality management of acute traumatic brain injured patients, the incidence of long lasting apallic syndrome full stage was accordingly 1,2 % of patients who received any kind of inpatient neurorehabilitation after TBI (**Table 4**). We could observe over the years and as it was clearly demonstrated in a prospective clinical study on class III evidence the functional impairment and fluctuation of mental cognitive functioning is best mirrored with the aid of the CRS score. Our prospective study on the prognostic reliability of CRS demonstrated that no patient with less than 20 points on day 40 reached a functional outcome of the Glasgow Outcome Scale (GOS) ,score 4 or 5. No patient with fewer than 10 points emerged from apallic syndrome (GOS 2) within one year [**18**]. A maximal score of 24 points of the 24 points CRS scale, respectively > +25 points ERBI (*Early Rehabilitation Barthel Index*), constitute the generally accepted cut-off criterion for ENNR [**20**,**42**].

**Table 4:** Early functional outcome (GOS) at time of discharge from rehabilitation institute (N= 258 TBI) with regard to GSC assessed at the beginning of TBI’s rehabilitation

GOS discharge	When GCS was assessed at the begin of rehabilitation				
	mild	moderate	severe	no data	total
5 no/minimal functional deficits %	45 39,1 %	8 19,5 %	-	15 18,1 %	68 26,4 %
4 moderate disability %	40 34,8 %	12 29,3 %	3 15,8 %	8 9,6 %	63 24,3 %
3 severe disability %	15 13,0 %	13 31,7 %	10 52,6 %	-	38 14,7 %
2 vegetative state VS %	-	-	3 15,8 %	-	3 1,2 %
1 dead %	-	1 2,4 %	1 5,3 %	-	2 0,8 %
Missing %	15 13,1 %	7 17,1 %	2 10,5 %	60 72,3 %	84 32,6 %
TBI patients %	115 100,0 %	41 100,0 %	19 100,0 %	83 100,0 %	258 100,0 %

**Fig. 1:**
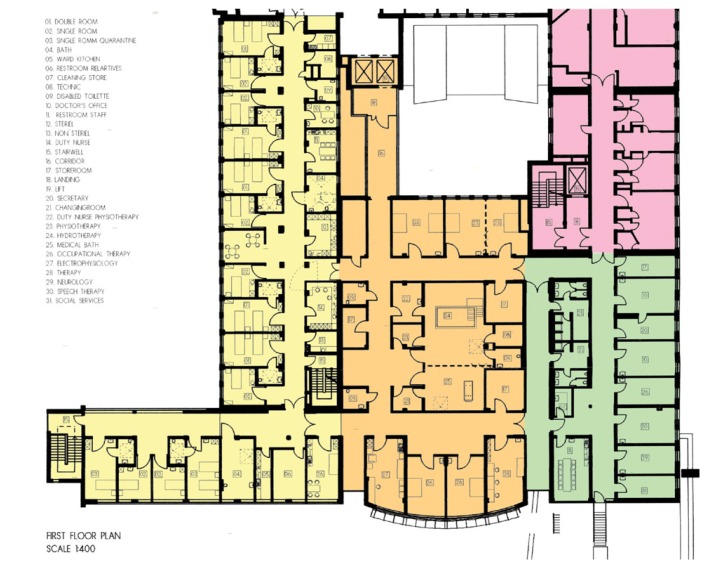
Floor plan of the first ever special unit of early neurorehabilitation in neurosurgery designed and established by the author to offer the patients neurosurgical know how and trans-disciplinary treatment in case of multiple injuries and secondary complication at any time.

**Figure 2:**
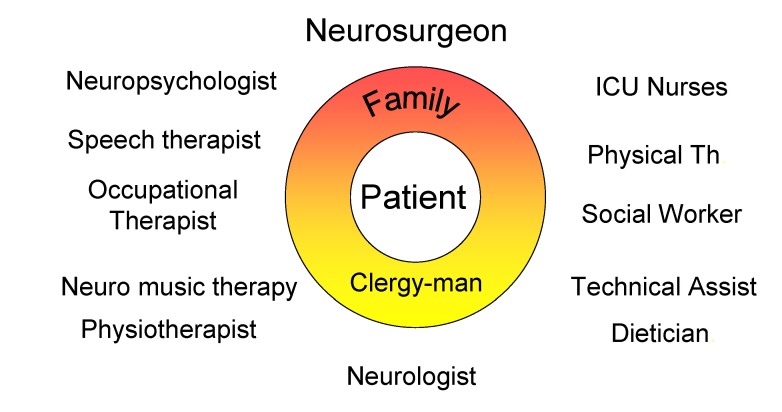
Inter- transdisciplinary team approach for early rehabilitation in neurosurgery

It has become evident over the last decade that progress and intensity of rehabilitative interventions and adequate medical caring insignificantly improved AS survival time (see **Table 3** and **4**, **Fig. 1** and **2**).

Up to now one of the most difficult things to achieve is a controlled environment for apallic patients that can be created to meet the special needs of this group of individuals (17). Oka and co-workers reported on their analysis of life prognosis of AS patients that were treated with aid of different medical care modalities [**36**]. The first group was treated state of the art at their special 50 beds CRC department for vegetative patients after traffic accidents from 1985 until 2003 (49 patients/year = total of 931 patient-years and 144.404 days of medical treatment) The yearly mortality rate was only 1.2% . On the contrary, for patients that have been discharged home to be cared in the community with financial support by the National Agency of Automotive Safety and Victims’ Aid (NASVA) between 1980 and 2003 the averaged annual mortality rate was 15.2%. The authors concluded that good life prognosis can be expected from special units for AS medical care and humanistic nursing providing a clean environment, an adequate primary nursing system, and easy and quick access of medical care to prevent and treat secondary and tertiary complications (9,23) Nevertheless the question of long-term survival is a matter of ongoing and controversial debate in the scientific literature and in the lay press. There are different opinions as to how long such patients could live and how long they do live in special rehabilitation centres, in nursing homes, or at their home with relatives. 

Jennett (17) stressed the fact that this may become more significant with the practice of limiting treatment once an *apallic state* is declared as *permanent* that is becoming more widely accepted, since long-term survivors are reported not to have benefited that impressively from modern methods of rehabilitative interventions and activating care, and some reports emphasized that care of a long survivor had been provided by an unsophisticated institution (Case III evidence). The survey of British clinicians views on management of patients in persistent vegetative state demonstrated a broad consensus among the doctors that treatment–limiting decisions are sometimes appropriate for patients in PVS, irrespective of whether they have experience of the condition or of the speciality to which they belong [**5**,**21**] The goal of the European survey, where doctors involved in the treatment of patients in „persistent vegetative state” were being interviewed, was to offer provisional orientation concerning the basic attitudes [**7**,**19**]. The hypotheses were confirmed that doctors from different European countries – as far as the decision of withdrawing or withholding treatment is concerned - are guided by different basic attitudes so that Lanzerath et al. concluded that decisions taken will depend on the guiding ethical assumptions [**19**], and by doing so decisions can only be derived at considering each individual case (at Class II evidence).

## Discussion

The overall incidence of new AS/VS full stage cases all aetiology is 0.5-2/100.000 population per year. About one third are traumatic and two thirds non-traumatic cases. Increasing figures for hypoxic brain damage and progressive neurological disease have been noticed. The main conceptual criticism is based on assessment and diagnosis of all different AS/VS stages based solely on *behavioural findings* without knowing the exact or uniform pathogenesis or neuropathologic findings and the uncertainty of clinical assessment due to varying inclusion criteria. No special diagnostics, no specific medical management can be recommended for class II or III AS treatment and rehabilitation. This is why *sine qua non* diagnostics of the clinical features and appropriate treatment of AS/VS patients of „*AS full, remission, defect and end stages*” require further professional training and expertise for doctors and rehabilitation personnel [**20**,**41**,**42**]. It has become apparent that due to important national differences it would not be advisable to recommend European guidelines on the management of such patients for all countries but to respect and follow national legal decisions [**41**]. In Germany, in 2006 for example, legal professions concluded after their conference on (passive) euthanasia (medicide, medically assisted suicide) that a considerable part of doctors and guardianship court like a former judge from the Federal High Court consider today determining of life supporting medical treatment as a criminal act of active euthanasia while taking into consideration at the same time the former ruling of the Federal High Court that withdrawal of assisted artificial nutrition remains exempt from punishment when in accordance with the patient’s avowed or presumably declared intention to die a natural death in the sense of dignity of man. They unanimously agreed that killing on request will remain an act of culpability That’s why we have to bring out special attention on the Hippocratic principles whereby the physician has never the permission and at least no right to finalize the life of his patient or another human life by active or passive measures. [**19**] This includes the physician ethical obligation not to miss necessary medical care and adequate supply of nutrition and liquid. To conclude, in following the Paris’ Declaration the active form of withdraw of nutrition and hydration in the apallic patient is to be classified as an active euthanasia neglecting the human rights so that the physician and/or his/her assistance would be sentenced for killing the patient in an helpless state incapable to decide or consent [**10**,**11**]. A crucial decision in the treatment of apallic patients is for the doctor to declare the date for ending the active rehabilitation treatment interventions with the consequence that the patient will be transferred to a special nursing home . If there is clinically no doubt as to the diagnosis of posttraumatic AS full stage or an early remission phases 1 to 2 (**Table 1**) and when repeated assessments unequivocally demonstrate the irreversibility of full stage AS – notwithstanding best possible intensive-care therapy and early neurorehabilitation measures - over more than 6 months in the case of hypoxic AS respectively more than 12 months - then, in consideration of the specific facts of the individual case, the question of further active rehabilitation programme for the patient can be raised. This decision must be made on the basis of concurrent diagnoses of two independent neurologists, one of them can be a neurosurgeon, with proven expertise in the field of neurological intensive-care medicine and rehabilitative treatment of AS patients [**23**-**37**]. Only on such basis of experienced physicians it will be permissible to end all unnecessary life-prolonging medical measures – or, more correctly, those that prolong the patient’s suffering – and thus enable the AS patient to die in a way compatible with his rights as a human being. Providing and securing basic physical care, pain relief, maintenance of respiratory potency, as well as the issues of artificial natural alimentation and sufficient supply of liquid intake via PEG probe are the fundamental obligations that are demanded from the doctors in terms of *basic therapy* that has to be continued in every case, even if there are no clinical signs of restoration of higher cerebral functioning in the AS full stage or an early remission phase. We strengthen this humanistic concept notwithstanding the fact that withdrawal of assisted nutrition and hydration is reported to be increasingly supported by some families, practitioners, scientific societies, medical hospitals and nursing homes, once the condition of an apallic full stage or an early remission state of minimally consciousness has been considered permanent [**13**,**14**]. „Withdrawal of nutrition and hydration from vegetative patients is a very difficult and sensitive issue for all people working with these patients and their relatives,” K. Andrews quotes [**4**]. There is the high moral value that must be attached to the love and engagement of the patient’s relatives that develop during the time of the AS disorder for the irreversibly unconscious or severely impaired patient, especially when the patient is being cared for at his home by his next of kin. In U.K., Holland, Belgium [**7**] one may submit the case to the High Court. Andrews’ expertise in United Kingdom by carefully reading his publications illustrates best state-of-the-art decision making for physicians and next of kin as based on the scientific literature and intensive discussions by taking also into account other European legal aspects [**3**, **4**]. There is nothing more to add but to claim for intensive experience and expertise in AS management before making any medical or rehabilitative decision [**41**]. This devoted care is an expression of human solidarity, and the helpless AS patient is existentially reliant on the dedicated protection and the appropriate degree of care by society to a special degree. The Hippocratic Oath and the occidental culture place the physician under a special obligation to do his utmost to uphold the life of the patient and also to respect his death when time comes. The enlightened person of this day and age rightfully expects that the physician in charge of his treatment feels committed to both the indivisible value of life as such, but also to respecting the patient’s will (witnessed and confirmed patient declaration). Enabling the patient to pass away by discontinuing artificial nutrition may not be misinterpreted as giving one person the permission to kill another as an active deed. The patient’s right to self-determination is binding for all concerned and must be respected by the physician even in cases when this will does not coincide with his (expert) medical opinion. In this context we have to make a god point again: It is imperative that the patient’s will should be contemporary and officially confirmed. We definitively do not agree in the face of increasing indices a tendency of lawyers and local district courts to accept and legalize also a non written but verbal and even emotional expressed patient’s will that is coming from relatives or friends in the context valid as the written and confirmed patient’s will. According to medical-legistic interpretation, euthanasia is understood as the deliberate induction of the death of a person; today, it is taken in the meaning of „mercy killing“, an act taken to end a life that will shortly be over [**10**,**11**]. In Austria and Germany, and indeed in the majority of European and non European countries, active euthanasia is subject to legal sanctions and is classified as a criminal act. Passive euthanasia, by contrast, might be considered as an act of mercy by the physician in the form of withholding treatment, i.e. he/she does not continue with all medical efforts and the basic care to actively maintain the life of the dying or soon-to-die patient [**18**,**19**]. This notwithstanding, the passive attitude does not relieve the physician and his assistants from their obligation to continue providing the patient with conscientious basic care and sufficient palliative and pain-relieving therapy on top of human attention not to come in danger to be accused for euthanasia because of starving the patient. When looked at „not feeding the patient” in this light, euthanasia is indeed not consistent with the Hippocratic Oath 

In case of a patient suffering from an AS in full stage or early remission phase when the provisional diagnosis is confirmed as hopeless to the doctors best knowledge than and only in this rare condition the physician who has taken the responsibility must have the right to decide the renunciation of maximal therapy in upcoming severe complications. This is the case of severe infectious states that are effective treatable only with a complicate mixture out of selected new potent antibiotics respectively in a case of severe, life threatening gastric or intestinal bleedings that can only be stopped with the aid of an emergency operation. There is no question about the doctor’s legal-ethical responsibility in respect of that final medical decision of renunciation of maximal therapy [**13**,**14**]. This decision can exclusively be taken only by the physician who knows the patient and his severe disturbances of higher cortical functioning over a longer time period. In that case only, this kind of medical decision might be conform to the Hippocratic principles [**18**].

## Conclusions

Major ethical objections against the ending of artificial nutrition and hydration on medical advice and approval should be interpreted as arguments in favour of the value of human life, independent of the status of brain functioning, and that every such person without consciousness and/or with severest cognitive impairments has exactly the same dignity and right to live as a person who is healthy from the mental-cognitive viewpoint. Passive euthanasia as already mentioned in the Hippocratic Oath will remain exempt from punishment only when in accordance with the patient’s official confirmed will of recent date to die a natural death in the sense of dignity of man. The decision to withdraw nutrition and liquid has a historical negative background and can cause for patients especially in remission stage feelings similar to those in starving. Full acceptable is the renunciation of maximal therapy and the pain therapy.
